# Hydroa, or Fever Blisters

**Published:** 1890-06

**Authors:** G. M. Pease

**Affiliations:** San Francisco, Cal.


### HYDROA, OR FEVER BLISTERS.

G. M. Pease, M. D., San Francisco, Cal.

By way of a postscript to Dr. McNeil's article on Natrum-mur., I would like to refer to the symptom of hydroa, or fever blisters. For a number of years I have closely watched these little things with the view of making them a point for differential diagnosis between Natrum-mur. and Rhus, and repeated clinical opportunities have to my mind settled the matter as follows:

On the *first* appearance the Rhus blister is clear or translucent, with a tendency to amber color. The Natrum-mur. blister is not as clear, but of a whitish or pearl color, nearly or quite opaque, and during its undisturbed progress does not change its color as soon as that of the Rhus.

The Natrum-mur. blister is more likely to be discrete, while the Rhus tends more to confluence or groups.

